# Lattice stability of ultrafast-heated gold

**DOI:** 10.1038/s41598-025-89470-7

**Published:** 2025-02-13

**Authors:** Sam Azadi, Justin S. Wark, Sam M. Vinko

**Affiliations:** 1 Clarendon Laboratory, Department of Physics, University of Oxford, Parks Road, Oxford, OX1 3PU UK; 2 STFC Rutherford Appleton Laboratory, Central Laser Facility, Didcot, OX11 0QX UK

**Keywords:** Phase transitions and critical phenomena, Laser-produced plasmas

## Abstract

First principle simulations within the framework of the finite-temperature density functional formalism predict the existence of nonthermal phase transitions in gold on ultrafast timescales with increasing electron temperature. The Gibbs free energy phase diagram as a function of electronic temperature indicates two solid-solid phase transitions of fcc$\rightarrow$hcp at an electronic temperature of 1.2 eV and hcp$\rightarrow$bcc at an electron temperature of 6.8 eV, while the ion lattice remains cold at zero temperature. We present a detailed analysis of the process of phonon-hardening in ultrafast-heated gold, using finite-temperature density functional perturbation theory simulations of the phonon spectra, the quantum thermodynamic phase diagram, and the thermoelastic properties.

## Introduction

Short-pulse light sources such as femtosecond optical lasers and x-ray free electron lasers (XFEL) can produce nonthermal and non-equilibrium systems from solids by rapidly driving electrons to high temperatures. Electron excitations generated by ultra-fast heating affect the electronic and magnetic structure of the system, its density of states, and lattice vibration spectra, but have also been shown to modify the ionic potential in surprising ways^1–12^. The resulting modified ionic forces can trigger non-equilibrium structural transitions in solids on picosecond timescales, even before any significant energy transfer between electrons and ions can occur^13–16^. The study of nonthermal phase transitions by laser irradiation has predominantly focused on semiconductors. Ultrafast melting under laser excitation was observed by time-resolved reflection second harmonic and reflectivity for GaAs^17^ and time-resolved x-ray diffraction for InSb^5^, Si^18^, Ge^13^, Bi^19^, and $\hbox {VO}_{2}$^20–22^. Ultrafast melting occurs in the surface layer of the material on a time scale of a few hundred femtoseconds depending on the laser energy.

Laser-induced phase transformations in covalent bond materials and semiconductors are predominantly studied theoretically via first principle methods and molecular dynamics simulations^16,18,23–28^. Semiconductors such as Si show a large phonon softening and instability of the transverse acoustic mode in the whole Brillouin Zone (BZ) with increasing electronic temperature. In contrast, phonons of metals, such as Al and Au, are predicted to harden and the melting temperature to increase as a result of increasing the electronic temperature. Our current understanding suggests that the size of the effect strongly depends on the interplay between the lattice and the electronic structure. It has been argued that, in Au, where the electronic DOS is dominated by the *d*-band, increases in electron temperature lead to a substantial change in the electronic DOS near the Fermi level interpreted as strong electron-phonon coupling, which in turn leads to a more pronounced phonon hardening^1^. In contrast, in a simple metal like Al, the electronic DOS remains nearly constant with temperature because of its free-electron-like behavior, leading to weaker electron-phonon coupling and less pronounced phonon hardening. The potential hardening of the phonon modes, which has also been suggested for other transition metals^1^, stabilizes the metallic lattice and delays the onset of the solid-liquid phase transition^2^.

Due to the complex behavior of d-band electrons, the electronic and structural properties of gold under extreme conditions have been studied extensively by theory and experiment^29–48^. However, there are disagreements among the theoretical and experimental results for the equation of state (EOS) and the physical behavior of gold under static and dynamic compression^30,41^. For example, while static pressure diamond anvil cell (DAC) measurements indicate that the fcc structure under ambient conditions continues to exist over a wide range of pressures^49^, first principles simulations predict a fcc$\rightarrow$hcp phase transition within a pressure range of 151 to 410 GPa^29,34,50,51^. Due to the polymorphism in gold, distinguishing between the fcc and hcp phases strongly depends on the theoretical framework used.

Femtosecond laser irradiation induces a complex series of processes in a solid prior to the complete dissipation of the energy, including melting, ablation, and pressure- or resolidification-velocity-driven phase transformations^14^. In contrast to semiconductors, where optical irradiation causes phonon softening resulting in nonthermal melting^5^, the melting process in metals is a thermal process requiring energy transfer from excited electrons to the ions^28,52^. Femtosecond electron diffraction measurements^2^ provided some evidence for phonon hardening in gold, which was connected to increased lattice stability at high electronic temperatures, supported by first-principles simulations^1,53^. In contrast, ultra-fast laser excitations of gold observed the heterogeneous coexistence of solid and liquid, and evidenced a heterogeneous-to-homogeneous melting transition^52^, which can be related to the softening of the atomic bond.

In this work, we show that the hot-electrons thermal pressure introduces a series of effects in metallic Gold at different electronic temperature regimes, via calculations of the thermodynamic phase diagram, phonon dispersion curve, and elastic moduli. We predict that laser radiation destabilizes the initial fcc structure of Au, leading to two solid-solid phase transitions of fcc$\rightarrow$hcp and hcp$\rightarrow$bcc at electronic temperatures of $\sim$ 1.2 and $\sim$ 6.8 eV, respectively. The bcc phase has a lower Debye temperature and consequently a lower melting temperature. In support of our argument, we investigate the relationship between metallic lattice stability and solid-solid nonthermal phase transitions in Au by simulating the equation of state (EOS) using density functional theory (DFT) and density functional perturbation theory (DFPT)^54^. The density functional formalism which was originally developed based on the non-uniform electron liquid model^55^ is an appropriate approach for the simulation of EOS of high-energy-density material (HED). Because the HED system behaves smoothly over distances that are large compared to the size of atoms and lattice parameters, the fluid description of electrons is a suitable picture.

## Results

**Fig. 1:**
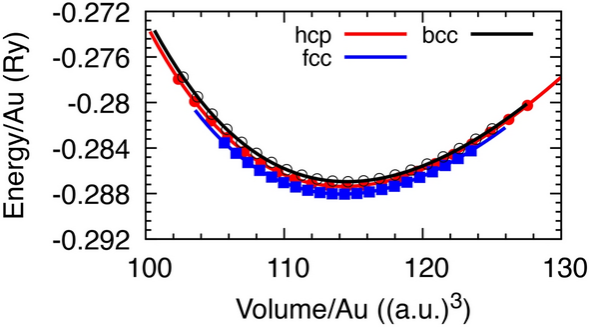
Total energy (data point) as a function of the volume interpolated by Murnaghan equation of state (fitted line) for fcc, hcp, and bcc structures. The equilibrium lattice parameters are $a_{fcc} = 7.701$, $a_{hcp} = 5.450$, and $a_{bcc} = 6.119$ Bohr, corresponding to the solid densities of $\rho _{fcc} = 19.331$, $\rho _{hcp} = 19.314$, and $\rho _{bcc} = 19.272$ g/$\hbox {cm}^3$, respectively. The energies were obtained at an electronic temperature of 60 meV. Energies are with respect to the energy of a single atom generated by the pseudopotential.

**Fig. 2:**
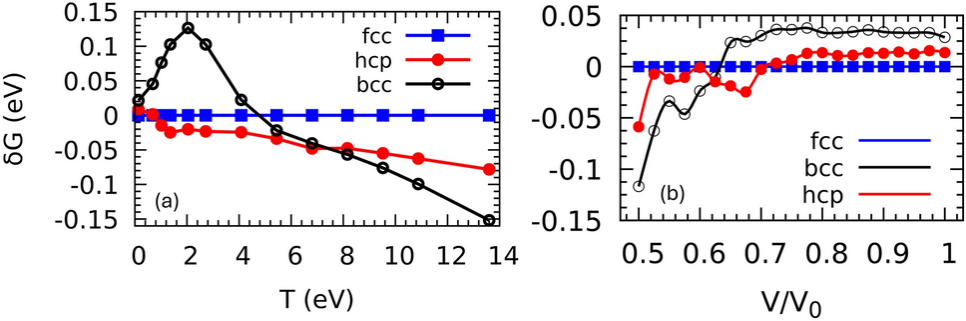
(**a**) Gibbs free energy of hcp and bcc phases with respect to the fcc phase as a function of electronic temperature. The hcp phase becomes stable above 1.2 eV, corresponding to a thermal pressure of 14 GPa, until 6.8 eV, corresponding to a thermal pressure of 226 GPa, where it transforms to bcc. (**b**) Gibbs free energy of the hcp and bcc phases with respect to the fcc phase as a function of compression. $V_0$ is the experimental equilibrium volume, 16.79 Å^3^^50^. The phase diagram predicts the isothermal phase transitions of fcc to hcp at compression $70\%$, corresponding to a volumetric pressure of 175 GPa, and hcp to bcc at compression $62\%$, corresponding to a volumetric pressure of 384 GPa.

**Fig. 3:**
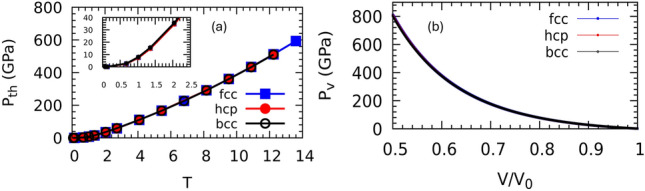
(**a**) Thermal pressure $\hbox {P}_\text {th}$ as a function of electronic temperature for the fcc, hcp, and bcc phases. The inset plot shows a rapid increase in $\hbox {P}_\text {th}$ at T$>1.5$ eV. (**b**) Volumetric pressure $\hbox {P}_\text {v}$ of fcc, bcc, and hcp structure as a function of compression. The pressures are obtained at an electronic temperature of 50 meV. $V_0$ is the experimental equilibrium volume, 16.79 Å^3^.

The equilibrium lattice parameters of the fcc, hcp and bcc structures at low temperatures and zero pressure were obtained using the Murnaghan equation of state (EOS), and are shown in Fig. 1. The optimized volume of the three structures gives a solid density of $\sim$19.3 g/$\hbox {cm}^3$, which agrees with the experiment. The Gibbs free energy *G* as a function of the electronic temperature phase diagram (Fig. 2) exhibits two phase transitions of fcc$\rightarrow$hcp and hcp$\rightarrow$bcc at electronic temperatures of $T_{fcc\rightarrow hcp}$=1.2, and $T_{hcp\rightarrow bcc}$=6.8 eV, respectively. According to the graph of the thermal pressure $P_{th}$ of the hot electrons versus the temperature of the electrons (Fig. 3), $T_{fcc\rightarrow hcp}$ and $T_{hcp\rightarrow bcc}$ are related to the thermal pressure of 14 and 226 GPa, respectively. The phase diagram of *G* as a function of isothermal compression shows two phase transitions of fcc$\rightarrow$hcp and hcp$\rightarrow$bcc at $70\%$, corresponding to volumetric pressure of 175 GPa (Fig. 3), and compression $62\%$, corresponding to volumetric pressure of 384 GPa (Fig. 3), respectively. It can be observed that the free energies of the fcc and hcp structures are similar for compressions up to $50\%$, but with the hcp phase becoming increasingly stable over the fcc, and the bcc phase becoming the most stable phase at compression higher than $60\%$.

To the best of our knowledge, the Gibbs free energy phase diagram of gold as a function of electronic temperature has not been studied before. However, there are extensive experimental and theoretical results on the effect of volumetric pressure on the structure of gold. Recently, ramp compression via laser ablation was used to dynamically compress gold to 690 GPa^30^. The polymorphism behavior of gold was studied by X-ray diffraction. The fcc phase was detected at 162 GPa, followed by a mixed region of fcc-bcc. Only the bcc phase was observed between 377 and 690 GPa. Although the fcc$\rightarrow$hcp phase transition was detected in an X-ray diffraction experiment at 248 GPa and 860 K using a heated diamond anvil cell^29^, other experiments using more advanced anvil designs observed the stability of the fcc phase up to 1065 GPa at room temperature^49,56^.

**Table 1:** Structure transitions of compressed Gold below room temperature predicted by previous DFT calculations using pseudopotential (PP), full-potential (FP), spin-orbit coupling (SO), all-electron linear combinations of Gaussian-type orbitals-fitting-function (LCGTO-FF), linear augmented plane wave (LAPW), and the machine learning accelerated canonical sampling (MLACS) methods with LDA and GGA XC functionals.

References	Structure transitions	Transition pressure (GPa)	DFT-method (XC)
^29^	fcc$\rightarrow$dhcp$\rightarrow$hcp	250, >600	PP-PAW (LDA)
^57^	fcc$\rightarrow$ABCACB$\rightarrow$dhcp$\rightarrow$hcp	620, 650, 780	PP-PAW (LDA)
	fcc$\rightarrow$ABCACB$\rightarrow$hcp	220, 480	FLAPW+lo (LDA)
^50^	fcc$\rightarrow$hcp	241	FP-LMTO (LDA)
	fcc$\rightarrow$hcp	200	FP-LMTO (GGA)
^51^	fcc$\rightarrow$hcp$\rightarrow$bcc	151, 400	FP-LMTO+SO (GGA)
^34^	fcc$\rightarrow$hcp	350	LCGTO-FF (LDA)
	fcc$\rightarrow$hcp	410	LCGTO-FF (GGA)
^37^	fcc$\rightarrow$hcp$\rightarrow$bcc	255, 475	FP-LMTO (LDA)
	fcc$\rightarrow$hcp$\rightarrow$bcc	240, 505	FP-LMTO (GGA)
	fcc$\rightarrow$hcp$\rightarrow$bcc	200, 520	FP-LMTO+SO (GGA)
^45^	fcc$\rightarrow$hcp$\rightarrow$bcc	500, 1000	PP-PAW(LDA)+(MLACS)
Present work	fcc$\rightarrow$hcp$\rightarrow$bcc	175, 384	PP-PAW (GGA)

From a computational point of view, there is disagreement among the predicted results for the stability and phase transition pressure. Density functional theory (DFT) using the local density approximation (LDA) and the generalized gradient approximation (GGA) exchange-correlation (XC) functional predicts that fcc transforms to the hcp phase at 350 GPa (LDA) or 410 GPa (GGA), which remains stable up to 1000 GPa^34^. The full-potential muffin-tin orbital (FP-MTO) DFT calculation shows a transition from fcc to hcp at 241 GPa (LDA) and 200 GPa (GGA)^50^. Another FP-MTO-DFT calculation with spin-orbit coupling and PW91 XC functional predicts two phase transitions of fcc-hcp and hcp-bcc at 151 and 400 GPa, respectively^51^. Other first principle simulations indicate a transition from the fcc phase to a double-hexagonal-close-packed (dhcp) phase at 232-250 GPa^29^, a series of stacking disordered phases above 390 GPa^57^. A detailed first principle calculation using the FP-MTO-DFT method suggests that at room temperature, compressed gold undergoes a sequence of phase transitions fcc$\rightarrow$hcp$\rightarrow$bcc at pressures 255 and 480 GPa, respectively^37^. Table 1 summarizes the phase transitions predicted by the previous first principle methods.

The volumetric pressure $\hbox {P}_\text {v}$ as a function of compression and the electronic thermal pressure $\hbox {P}_\text {th}$ as a function of the electronic temperature are illustrated in Fig. 3. Our calculated values for the elastic constants $C_{ij}$ of the fcc phase at T=50 meV are $C_{11}=194$, $C_{12}=167$, and $C_{44}=39$ GPa, which are in good agreement with the experimental values at room temperature $C_{11}=202$, $C_{12}=170$, and $C_{44}=45$ GPa^37^. Our results indicate that the electronic thermal pressure $\hbox {P}_\text {th}$ increases with electronic temperature (Fig. 3). The behavior of the electronic thermal pressure as a function of electronic temperature strongly depends on the density of the electronic states. The DFT-LMTO method with LDA XC functional was used to study the effect of electronic temperature on thermal pressure in Au and Pt^58^. We found that $\hbox {P}_\text {th}=2.5$ GPa for Au at T = 0.68 eV, which agrees with their results. They observed that at 2000 K the $\hbox {P}_\text {th}$ of Pt is 0.85 GPa, while $\hbox {P}_\text {th}$ of Au is much smaller, and even at 4000 K the $\hbox {P}_\text {th}$ of Au becomes 0.26 GPa^58^.

**Fig. 4:**
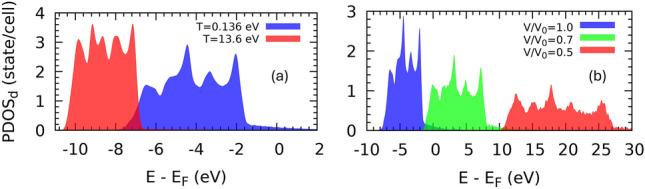
(**a**) Projected DOS for d-band of fcc phase at two different electronic temperatures. Increasing the electronic temperature decreases the bandwidth and shifts the band to lower energies. (**b**) Projected DOS for the d-band of the fcc phase at different densities. Increasing the density by compression enlarges the bandwidth and moves the band to higher energies.

The electronic density of states (DOS) in the vicinity of the Fermi energy $E_F$ is dominated by the *d*-band. The projected DOS for the d-band of the fcc phase indicates that increasing the electronic temperature reduces the bandwidth and shifts the band to lower energies (Fig. 4). Loẅdin population analysis, which was carried out alongside projected DOS calculations, shows that the localized charges of *d*-orbitals decrease due to thermal excitation and, consequently, the smaller on-site Coulomb repulsion with respect to the kinetic energy of excited electrons narrows the bandwidth. Volumetric compression reduces *d*-band localization by bringing the atoms closer to each other, resulting in band overlap and extending the bandwidth (Fig. 4). Unlike the electronic temperature, volumetric compression shifts the d-band to higher energies. The projected DOS of the hcp and bcc phases shows similar behavior.

At low electron temperatures $\sim$0.1 eV ($\sim 10^3 K$) mainly the *s*-band electrons behave as an excited free-electron gas. At higher electronic temperatures $\sim 1$ eV ($\sim 10^4 K$) the *d*-band electrons can be excited and contribute to thermophysical properties. The projected DOS (Fig. 4) shows that the edge of the *d*-band electrons is located $\sim$1.4 eV below the Fermi energy, which explains the rapid increase in $\hbox {P}_\text {th}$ at the same electronic temperature. The contribution of electronic thermal pressure in measuring the pressure in high-pressure high-temperature experiments is expected to be small or large depending on the density of the states of the studied system.

**Fig. 5:**
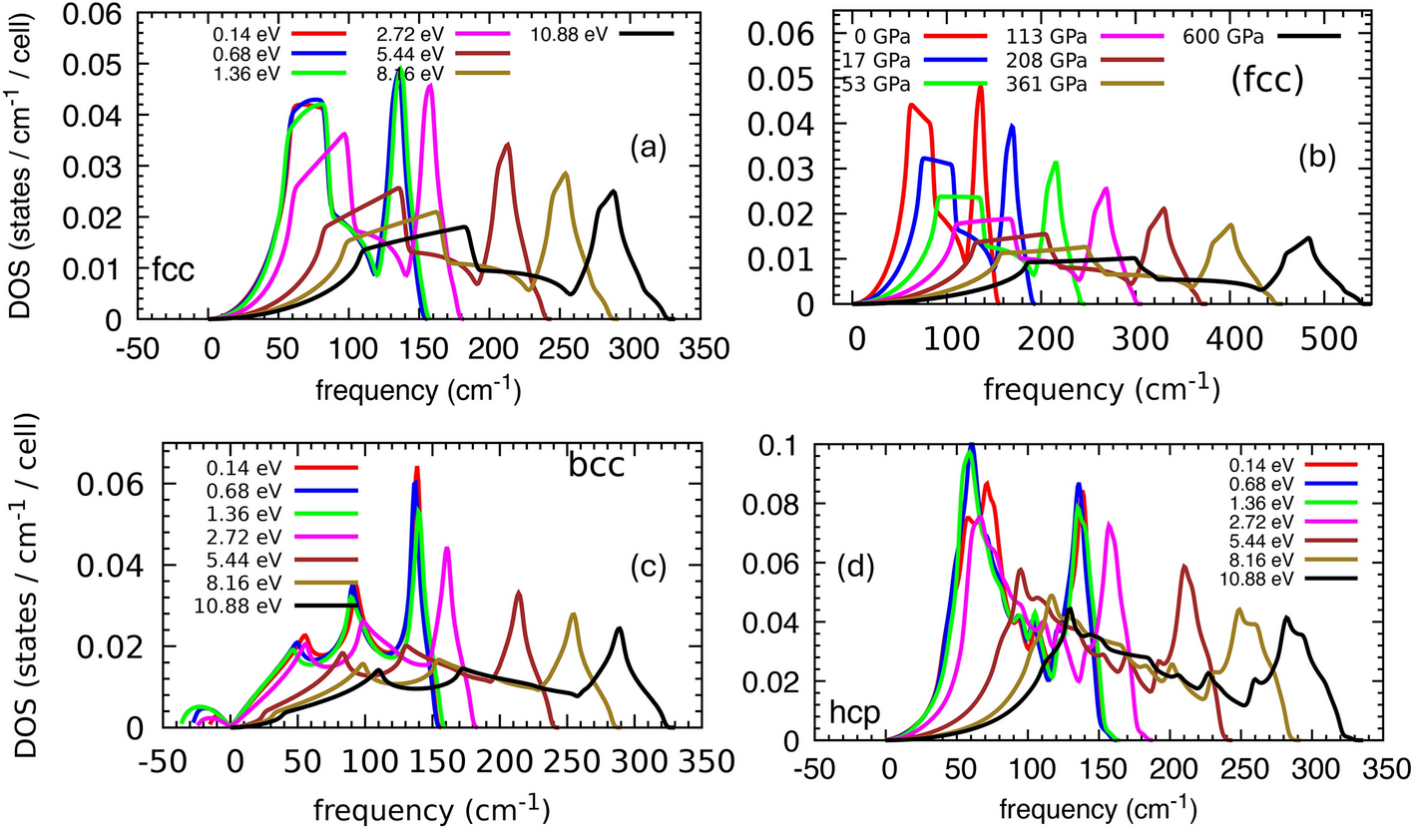
Phonon DOS of fcc structure as a function of electronic temperature (**a**) and volumetric compression (**b**). Phonon DOS of the bcc (**c**) and hcp (**d**) structures at different electronic temperatures up to $\sim$11 eV. The fcc and hcp phases are dynamically stable at all the studied electronic temperatures, whereas the bcc shows imaginary phonons at temperatures below 5 eV. The phonon hardening in fcc and hcp at temperatures below 1.4 eV ($\hbox {P}_\text {th} <15$ GPa) is very small.

**Fig. 6:**
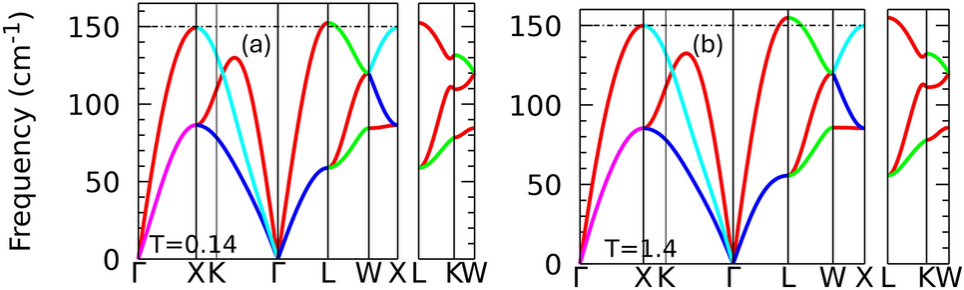
Phonon dispersion curve of fcc phase at T=0.14 (**a**), and 1.4 (**b**)eV. Comparison of the dashed horizontal line in two plots indicates negligible phonon hardening while the electronic temperature is increased by an order of magnitude.

Both the fcc and the hcp structures are thermodynamically unstable at temperatures above 6.8 eV, but dynamically stable without any sign of imaginary phonons in their phonon dispersion curves (Fig. 5). The phonon density of states (DOS) of the fcc phase shows a phonon hardening by increasing the electronic temperature and can be expounded as a sign of metallic lattice stability and the enlargement of the melting temperature due to laser radiation^1^. However, according to the Gibbs phase diagram (Fig. 2), this phase is only stable at electronic temperatures below $\sim$1.2 eV where the phonon hardening due to electronic temperature is insignificant. The hardening of the phonons, which is introduced by increasing the electronic temperature by an order of magnitude from 0.14 to 1.4 eV, is very small (Fig.6). The thermal pressure $\hbox {P}_{th}$ increases by 14 GPa within the same electronic temperature range of 0.14 to 1.4 eV, while $\hbox {P}_{th}$ becomes almost three times larger by increasing the temperature from 1.4 to 2.1 eV (Fig. 3). Volumetric compression also causes phonon hardening. Figure 5 illustrates the phonon hardening in the fcc phase as a function of external pressure. The phonon DOS of the hcp and bcc phases as a function of compression also show a similar behavior.

We have not included the ionic temperature in our phase diagrams and phonon spectra analysis. Our results describe a situation where the electronic temperature is not transferred to the ions and where electrons and phonons are not thermalized. It was shown that the ionic temperature can affect the fcc$\rightarrow$hcp$\rightarrow$bcc phase transitions in volumetric compression^37^. The quasi-harmonic approximation was used to include the thermal contribution caused by the ionic temperature in the Au phase diagram, and it was found that the fcc phase can transform directly to bcc at ionic temperature $> 2000$ K and volumetric pressure $> 380$ GPa^37^. Hence, the stability of the hcp phase, and the possibility of observing it in an experiment, depend on the ionic temperature.

**Fig. 7:**
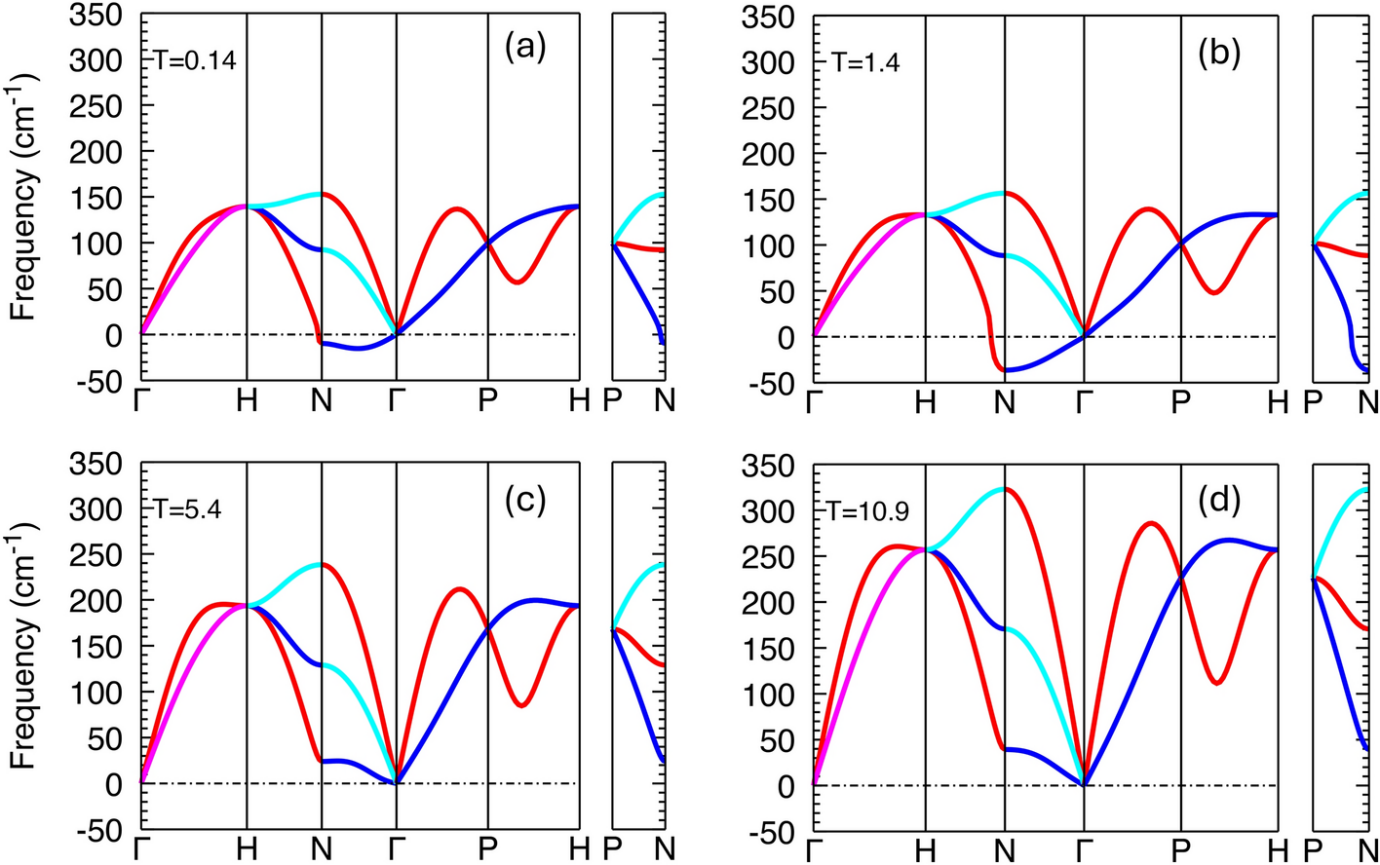
Phonon dispersion curve of bcc phase at T=0.14 (**a**), 1.4 (**b**), 5.4 (**c**), and 10.9 (**d**) eV. Comparison of the dashed horizontal line in two up-panel plots indicates phonon softening introduced by electronic temperature. The disappearance of imaginary modes and the hardening of phonons by increasing the electronic temperature can be observed from the plots in the bottom panel.

The phonon spectra indicate that the effect of increasing the electronic temperature by laser radiation depends on the crystal structure of the sample. The phonon dispersion curve of the bcc phase exhibits increasing imaginary phonons and phonon softening, within the same temperature range of 0.14 to 1.4 eV (Fig.7). The phonon dispersion curves of bcc show an anomalous dip in the lowest branch of the transverse acoustic modes along the $\Gamma -\text {N}$ direction at low electronic temperatures. This anomaly results from a qualitative change in the electronic screening because of the modification of the electron-phonon coupling with electronic temperature. Analysis of bcc phonon spectra at low temperatures shows that dynamical instabilities, which lead to imaginary frequencies, occur in a localized region in the ${\textbf {q}}$-space. This anomaly in reciprocal space is driven by the electronic structure and can result in Kohn anomalies, i.e., dips in the phonon band structure due to screening of the electronic state near the Fermi energy involved in electron-phonon coupling. The size of the Kohn anomaly is affected by the Fermi surface and the ${\textbf {q}}$ functionality of the electron-phonon coupling matrix. If the Fermi surface adopts a flat area with nesting vector ${\textbf {q}}_\textrm{ano}$, there could be a large screening of the potential perturbation, leading to atomic displacements in ${\textbf {q}}_\textrm{ano}$ and a softening that is localized in reciprocal space. The size of the Kohn anomaly is sensitive to electronic temperature, and increasing the temperature can reduce the sharpness of the Fermi surface, weakening the anomaly. This effect can be observed from the phonon spectra of bcc at higher electronic temperatures (Fig. 7).

**Fig. 8:**
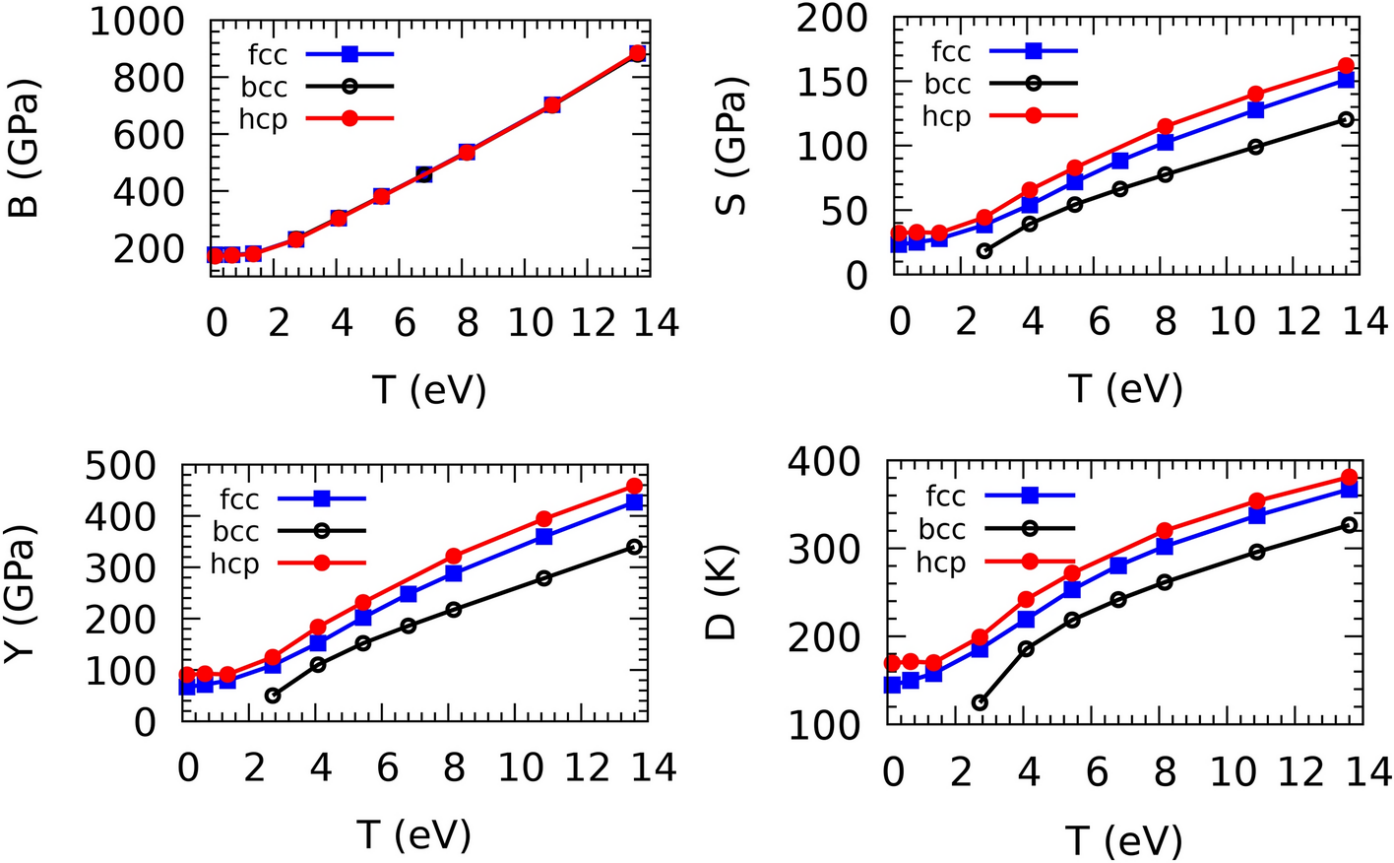
Bulk (B), Shear (S), Young (Y) modulus, and Debye (D) temperatures of fcc, hcp, and bcc structures as a function of electronic temperature. The experimental values for B, S, and Y at ambient conditions are 220, 27, and 78 GPa, respectively.

The nature of phase transitions driven by irradiation can be understood by analyzing the behavior of the elastic modulus as a function of the electronic temperature. Similarly to $\hbox {P}_{th}$, the Bulk (B), Shear (S), Young (Y) modulus, and Debye (D) temperature of the structures of fcc, hcp, and bcc increase sharply with an electronic temperature above 1.4 eV. However, at low temperatures, <1.4 eV, the temperature dependence of the elastic moduli of fcc and hcp is negligible compared with T>1.4 eV. Because of imaginary phonons for bcc, the elastic moduli were not calculated at low temperatures. The difference between the B moduli of the fcc and hcp phases is negligible, but the S and Y moduli of the hcp phase are slightly larger than those of the fcc. This difference suggests that the phase transition fcc-hcp at $\sim$ 1.2 eV is driven by the shear forces introduced by $\hbox {P}_{th}$. The Y- and S-moduli and the atomic packing factor of bcc are smaller than those of the hcp phase. As can be seen in Fig. 8, although the electronic temperature causes phonon hardening, the Debye temperature and consequently the melting temperature of the bcc phase are lower than those of the fcc and hcp phases.

## Discussion

Our results are of interest in the context of the recent observation of experimental discrepancies in phonon hardening in Au with increasing electron temperature^2,52,59^. The interpretation of the experimental results, which involve very thin Au samples, has so far assumed that the fcc phase remains the stable phase until the sample is driven to melt. However, as we have shown above, the phase diagram as a function of electronic temperature is expected to be far more complex, and in terms of the sequence of phases, it seems similar to that of Au under compression. We note that our theoretical approach does not allow for the direct study of the kinetics or dynamics of Au samples under ultrafast electron heating. However, the presence of hcp and bcc phases with lower thermodynamic free energies for electronic temperatures between 1-7 eV suggest that the presence of solid-solid phase transitions may affect the timescales within which fcc order is lost and would do so in a way that is strongly dependent on the irradiation intensity and on the timescale of electron-phonon coupling, given the important role ion temperature plays in phase transformations. Such effects should therefore be considered when melting temperatures and timescales are inferred from experimental data showing loss of fcc crystalline ordering.

Although both thermal and volumetric pressures introduce phonon hardening in all of the structures studied, they have opposite effects on the electronic structure and the density of states. The analysis of the projected density of states indicates that thermal excitations shift the d-band, which dominates the electronic DOS near the Fermi energy, to lower energy and lessen the bandwidth, whereas volumetric compression shifts the d-band to higher energies and enlarges the bandwidth. The ultrafast laser radiation reduces the localized charge density of the d-band by increasing the kinetic energy of electrons and consequently decreasing the bandwidth. Static pressure causes the band overlap by reducing the atomic distance and increasing the density and therefore the bandwidth widens.

Ultra-fast electron heating has a double-sided effect on the stability of the metallic bonding of Au. On the one hand, hot-electron thermal pressure leads to phonon hardening and an increase in the melting temperature. On the other hand, $\hbox {P}_\text {th}$ destabilizes the initial structure, which can lead to solid-solid phase transitions possibly affected by coherent acoustic phonons. The stable bcc phase has smaller Young and Shear moduli, a lower Debye temperature, and consequently a lower melting temperature. In this work, we have only considered the simple structures of fcc, hcp, and bcc, and we cannot rule out the possibility of the appearance of low-symmetry amorphous structures with even lower melting temperatures. Hence, without taking into account the stability of new phases, it cannot solely be concluded that femtosecond laser radiation makes the metallic bonding in gold stronger or weaker.

Our static thermodynamic phase diagram predicts the solid-solid phase transition, but the mechanism behind it is intricate due to the complex dynamics of the system during the phase transition. Addressing this process in detail is beyond the scope of the current work, but we do wish to highlight that the ultrafast heating of electrons in gold can introduce two types of lattice dynamics: one due to electron-phonon coupling, and another due to coherent acoustic phonons^60–62^. The two occur on different time scales. The thermal energy transfer due to electron-phonon coupling has been widely studied by theory and experiment, and has been considered to be the main source of the effect of hot electrons on the lattice dynamics. However, the effect of coherent acoustic phonons on the lattice dynamic is less discussed. Coherent phonon dynamics refers to the collective oscillations of atoms in a crystal lattice that occur in phase (coherently) across the system. Unlike incoherent phonons, which represent random atomic vibrations and contribute to heat, coherent phonons involve a well-defined vibrational mode excited coherently, which can be stimulated by ultrafast laser pulses. Monoatomic FCC-gold has no optical phonon branches, but only three acoustic phonon branches of one longitudinal acoustic and two transverse acoustics. The acoustic phonon frequencies go to zero near $\Gamma$ and increase roughly linearly away from $\Gamma$ (Fig. 6). Immediately after absorption of energy from laser pulses by electrons near the Fermi energy, the electron gas is at a much higher temperature than the lattice due to large difference between their specific heat capacity. The jump in hot-electron pressure at a specific electronic temperature (Fig. 3) can create an acoustic strain pulse if the electronic temperature is high enough^63^. The strain pulse can generate a rapid contraction (changing the Au-Au bond length) of the lattice that can be observed from phonon hardening (Fig. 6). These strain pulses propagate as coherent acoustic phonons at the speed of sound ($\sim$ 10-5 m/s) in gold. The strain pulse can be generated directly by the hot-electron pressure occurring almost instantaneously after laser excitation ($\sim$ 10-100 fs). The lattice responds coherently to this impulsive stress without requiring significant energy transfer or equilibration. These strain pulses are of interest as they may facilitate a solid-solid phase transition, depending on the strain amplitude. It should be noted that phonon hardening can coexist with coherent phonon phenomena, but they are conceptually distinct. Phonon hardening is an effect on the frequency value itself, whereas coherent phonons describe a collective oscillation at (or near) that frequency. Coherent acoustic phonons are not directly visible from our static phonon dispersion curve, because they are a non-equilibrium, time-domain phenomenon. However, they can be verified by measuring their oscillation frequency and then matching it to the static equilibrium branch in the dispersion, which provides all the possible vibrational modes. This comparison tells us which branch and wave vector were involved in coherent phonons.

For a full comparison between simulation and experiment, the effect of laser radiation on the gold metallic surface, which can be investigated using a large-scale *ab-initio* molecular dynamic simulation, has to be considered^64^. Ultrafast laser pulses can produce a non-equilibrium hot electron gas on the metallic surface, which can be observed in IR reflection and electron emission^65,66^. Our simulations suggest that one process that can happen on a time scale shorter than the cooling time of the electron gas $\sim 1ps$, is the lattice expansion. This effect is caused by the contribution of the hot electron gas pressure to the elastic constants, the sound velocity, and the phonon spectra by the effective electron force proportional to $\nabla T_{el}$. Hence, there is a possibility of lattice deformation near the metallic surface while the ions are still cold. Distinguishing between lattice deformation (or expansion) and heating is possible by comparing the shift and intensity variation of Bragg peaks with the observation of Debye-Waller factor. Depending on the electron temperature, the initial elastic deformation can introduce a plastic deformation.

Our results thus indicate that the evolution of nonthermal metallic systems can be highly complex, with a strong dependence on the detailed temporal interplay between the heating of the electrons, loss of electronic order, electron-phonon coupling, and ion motion. This suggests that the heating process itself may play an important role in the interpretation of experiments, since in practice electron heating and electron thermalization are not instantaneous. Furthermore, homogeneous heating of metallic samples using optical lasers limits targets to thicknesses of a few tens of nanometers^59^, and surface effects can further complicate the evolution of the samples on picosecond timescales.Depending on the material and regimes of interest, the difference between what is being modeled and what is being measured may well be significant, but it is challenging to predict *a priori*. Understanding and bridging this gap will be critical to further our understanding of how real materials behave in extreme, non-equilibrium conditions. One approach would be to seek to extend our simulations to match realistic experimental conditions. This remains a substantial theoretical and computational undertaking, and although some encouraging recent work has been done in this direction^6,15^, it is unlikely to be feasible in the near term. An alternative approach is to design experiments that better constrain the system and thus are simpler to model with the tools available today. Importantly, moving from optical laser heating to femtosecond x-ray heating, now possible with X-ray free-electron lasers (XFELs)^67^, would allow us to create much larger, micrometer-sized bulk samples. Furthermore, X-ray isochoric heating can create homogeneous electron temperatures in the 1-10 eV range within a cold ion lattice and can even control the detailed structure of the hot-electron distribution^68^. Similarly, hard X-rays are ideally suited to probe the ultrafast dynamics of both electrons and ions. Combining the two would thus allow us to better constrain the initial nonthermal conditions and to measure the subsequent evolution of the electron and ion systems independently. With the advent of novel two-color pump-probe capabilities that combine soft and hard x-rays, such experiments may soon be within reach.

The DFT results presented in this work were obtained by using two main approximations of the exchange-correlation functional and the frozen core pseudopotential (PP). Altering the exchange-correlation functional may affect the results quantitatively. Unfortunately, there is no systematic way to quantify the errors introduced by the choice of exchange-correlation functional. Within the frozen core PP approximation, it is assumed that the spectrum and spatial distribution of the valence electrons under the effect of temperature and pressure are negligible, which is a valid assumption for low temperature and pressure. At high temperatures, the core electrons are excited and therefore the potential of the core is modified. This error, which depends on the temperature, can be reduced if the number of electrons in the core is decreased, as we used a small core PP for gold. One systematic way to accurately analyze this error is to compare all-electron results with PP, which is a separate work. Last but not least, we assumed that the effect of electronic temperature on the XC potential is negligible, and that using a zero-temperature parameterization of the XC functional is a valid approximation.

## Methods

Electronic structure, lattice dynamics, and thermoelastic properties were obtained using finite-temperature density functional theory (DFT)^69–72^ and density functional perturbation theory (DFPT), as implemented in the Quantum Espresso package^73,74^. We used the revised Perdew-Burke-Ernzerhof generalized gradient approximation (PBEsol)^75^ for the exchange-correlation (XC) functional. We considered fcc, hcp, and bcc crystal structures of Au at electronic temperatures of up to 14 eV, using a kinetic energy cutoff of the plane-wave basis set of 200 Ry and an augmentation charge energy cutoff of 1600 Ry. The electronic temperature was controlled by the Fermi-Dirac distribution function, and the number of bands was increased by the electronic temperature so that several empty bands were always included in the conduction band. The calculations were carried out using a (32, 32, 32) $\textbf{k}$-grid and a (8, 8, 8) $\textbf{q}$-grid for DFT and DFPT, respectively. The projector-augmented wave (PAW) pseudopotential^74,76^ was generated using the scalar-relativistic calculation with 19 valence electrons.

The pressure introduced by the thermal excitation of electrons is called the electron thermal pressure $P_{th}$, and is written as:

1 $$\begin{aligned} P_{th} (V,T) = -\left( \frac{\partial \Delta F_{el}(V,T)}{\partial V}\right) _T \end{aligned}$$

where $\Delta F_{el}(V,T) = F_{el}(V,T) - F_{el}(V,T=0)$ is the thermal contribution to the electronic free energy $F_{el}(V,T)=E_{el}(V,T) - TS_{el}(V,T)$, where the $E_{el}$ and $S_{el}$ are the total ground state electronic energy and the entropy, respectively. The electronic density of states $n(\epsilon ,V)$ can be used to obtain the total energy and entropy:

2 $$\begin{aligned} E_{el}(V,T)= & \int _{-\infty }^{\infty } d \epsilon \; n(\epsilon ,V) \; \epsilon \; f(\epsilon ,T) \end{aligned}$$

3 $$\begin{aligned} S_{el}(V,T)= & \int _{0}^{T} \frac{c_v(V,T)}{T} dT \nonumber \\= & -k_B \int d \epsilon \; n(\epsilon ,V) \; [flnf+(1-f)ln(1-f)] \end{aligned}$$

where $f(\epsilon , V)=[1+\exp (\epsilon - \mu )/k_BT]^{-1}$ is the Fermi-Dirac distribution function, $k_B$ is the Boltzman constant, and $c_v=(\partial E_{el}/\partial T)_{V}$ is the electronic specific heat.

## Data Availability

All data generated and analysed during this study are available from the corresponding author on reasonable request.
